# Inappropriate Usage of Dietary Supplements in Patients by Miscommunication with Physicians in Japan

**DOI:** 10.3390/nu6125392

**Published:** 2014-11-26

**Authors:** Tsuyoshi Chiba, Yoko Sato, Tomoko Nakanishi, Kaori Yokotani, Sachina Suzuki, Keizo Umegaki

**Affiliations:** Information Center, National Institute of Health and Nutrition, 1-23-1 Toyama, Shinjuku-ku, Tokyo 162-8636, Japan; E-Mails: satoyoko@nih.go.jp (Y.S.); nakanisi@nih.go.jp (T.N.); yokotani-k@swu.ac.jp (K.Y.); sachina-s@nih.go.jp (S.S.); umegaki@nih.go.jp (K.U.)

**Keywords:** dietary supplements, patients, treatment of diseases, adverse effects

## Abstract

Recently, people have used dietary supplements not only for nutritional supplementation, but also for treatment of their diseases. However, use of dietary supplements to treat diseases, especially with medications, may cause health problems in patients. In this study, we investigated use of dietary supplements in patients in Japan. This survey was conducted from January to December 2012, and was completed by 2732 people, including 599 admitted patients, 1154 ambulatory patients, and 979 healthy subjects who attended a seminar about dietary supplements. At the time of the questionnaire, 20.4% of admitted patients, 39.1% of ambulatory patients, and 30.7% of healthy subjects were using dietary supplements, which including vitamin/mineral supplements, herbal extracts, its ingredients, or food for specified health uses. The primary purpose for use in all groups was health maintenance, whereas 3.7% of healthy subjects, 10.0% of ambulatory patients, and 13.2% of admitted patients used dietary supplements to treat diseases. In addition, 17.7% of admitted patients and 36.8% of ambulatory patients were using dietary supplements concomitantly with their medications. However, among both admitted patients and ambulatory patients, almost 70% did not mention dietary supplement use to their physicians. Overall, 3.3% of all subjects realized adverse effects associated with dietary supplements. Communication between patients and physicians is important to avoid health problems associated with the use of dietary supplements.

## 1. Introduction

With the rapid increase in the senior population in Japan, chronic diseases associated with aging, such as diabetes mellitus, cardiovascular disease, hypertension, osteoporosis, and cancer have become a widely recognized social issue. Against this background, an increase in health consciousness prompts people to use dietary supplements to maintain health and prevent diseases. Most people use vitamin or mineral supplements, whereas other herbal extracts (e.g., blueberry, coleus forskohlii, ginkgo, or green tea) and ingredients (e.g., collagen, catechins, fish oil, glucosamine, hyaluronic acid, and isoflavones) are also popular in Japan. People tend to believe that dietary supplements are as safe as food and as beneficial as medicine.

The beneficial effects of food and its nutrients and other ingredients have been recognized for a long time. Previously, people obtained nutrients and other ingredients only as foods, such as vegetables, fruits, fish, meat, tea, and other items. Over time, manufacturers learned to extract and condense some of the specific nutrients or ingredients in food and offer them as dietary supplements in the form of tablets, capsules, or powders. The concentrated ingredients in dietary supplements carry not only the benefits but also the risk of toxicity, interaction with drugs, and adverse reactions compared with the ingredients in whole foods [1]. However, manufacturers tend to emphasize key characteristics of their products and promote sales using attractive claims. In addition, in some cases, manufacturers claim that medicines are more likely than dietary supplements to cause side effects, because medicines are synthetic compounds, whereas dietary supplements are made from natural substances and thus safe and suitable for everybody. Currently, there is insufficient evidence that dietary supplements improve disease in humans, and if patients turn to dietary supplements instead of medicines, health problems might occur. Indeed, adverse effects caused by dietary supplements, especially hepatotoxicity associated herbal supplement use, are reported worldwide [2,3,4].

Regulation of dietary supplement in Japan is more complicated compared to other countries such as the USA or European countries. In 1991, the Ministry of Health, Labour and Welfare set up the Food for Specified Health Uses to provide people with accurate health information about foods. The current Japanese system for regulation of health foods is called Food with Health Claims and is made up of two categories: (1) “Food with Nutrient Function Claims” and (2) “Food for Specified Health Uses”. Most of “Food for Specified Health Uses” products are the form of regular food, such as tea, beverage, yogurt, and flakes. On the other hand, except for “Food with Health Claims”, laws for dietary supplements are not defined in Japan. This means that most dietary supplements on the market are considered the same as other foods, even if they are in the form of capsules or tablets [5,6].

Consumers tend to have only a vague understanding that dietary supplements are different from medicines, and some consumers use dietary supplements as medicines to treat specific diseases in Japan. Several reasons contribute to this inappropriate use of dietary supplements. First, there is no clear, official definition of dietary supplements in Japan. Because of this, many dietary supplements claim to treat specific diseases, especially cancer, even though such claims are illegal in Japan. Secondly, dietary supplements available as capsules or tablets look like medicines and thus are often thought to be as effective as medicines. Thirdly, consumers do not understand the properties of dietary supplements. Physicians are concerned about the use of dietary supplements by their patients, because of the possibility of dietary supplement–drug interactions [7]. In particular, dietary supplements may interact with some medicines as well as affect anaesthesia and bleeding during surgery [8].

Dietary supplements are helpful to complement nutrition in not only healthy subjects but also patients. However, if patients use dietary supplements to treat diseases without consulting physicians, it may cause health problems. This study used a self-administered questionnaire to clarify awareness and use of dietary supplements among patients in Japan.

## 2. Methods

### 2.1. Subjects

Subjects included 2732 people, who either attended health food seminars (Iwate, Ibaragi, Fukushima, Tokyo, Kanagawa, Shizuoka, Gifu, Wakayama, Fukui, Okayama, and Ehime), visited pharmacies (Tokyo, Shizuoka, Okayama), or were admitted to hospitals (Iwate, Tokyo, Saitama, Chiba, Shizuoka, Aichi, Okayama, Fukuoka, Nagasaki, Miyazaki, Saga, Kumamoto, Kagoshima) from January to December 2012. To clarify the recognition and use of dietary supplements among patients, we asked all subjects about their medical status and divided into three categories, admitted patients, ambulatory patients, and healthy subjects. Healthy subjects were defined as people who were not hospitalized or making regular visits to the hospital. This study was conducted according to the guidelines laid down in the Declaration of Helsinki and all procedures involving human subjects/patients were approved by the Research Ethics Committee of the National Institute of Health and Nutrition and each participating institute.

### 2.2. Definition of Dietary Supplements

Dietary supplements are well defined in the USA and European countries, because they are regulated by law, but they are not regulated in Japan. Dietary supplements were usually recognized in the form of capsules, tablets, powders, or liquid. However, some dairy or soybean products are also produced as dietary supplements, even if they are in the form of regular foods. Thus, we did not define a specific form for dietary supplements in this survey. Dietary supplements were defined as foods, other than vegetables and fruits that subjects thought would have beneficial effect on their health.

### 2.3. Questionnaire

The questionnaire included demographic characteristics (sex and age), information on use of supplements, awareness of dietary supplements (safety, price, effectiveness, substitute for medicines, and co-administration with medicines), purpose (maintenance of health, nutritional support, prevention of disease, treatment of disease, beauty, no specific purpose), number of dietary supplements used, realization of beneficial and adverse effects, and type and their situation of medications. In addition, the questionnaire asked whether subjects informed their physicians of their use of dietary supplements.

### 2.4. Statistical Analysis

Differences in demographic characteristics or supplement use among admitted patients, ambulatory patients, and healthy subjects were tested using the χ^2^ test or Kruskal-wallis test with Bonferroni correction. Univariate analysis for the association of supplement use with various variables in the patients and healthy subjects was done using the χ^2^ test. Multivariable analysis was also done using the logistic regression analysis adjusted for sex and age. *P* values less than 0.05 in χ^2^ test and 0.0167 in Kruskal-wallis test were considered significant. A statistical analysis was performed using SPSS 18.0J for Windows (IBM Co., Armonk, New York, NY, USA).

## 3. Results

### 3.1. Characteristics

Characteristics of all subjects (*n* = 2732) are shown in Table 1. More than half of subjects were female, and ages ranged from younger than 20 years to older than 80 years. Among all subjects, 21.9% were admitted patients, 42.2% were ambulatory patients, and 35.8% were healthy subjects.

**Table 1:** Characteristics of each group.

	Healthy Subjects	Ambulatory Patients	Admitted Patients	Total	*p*-value
**Number of Subjects (%)**	979 (35.8)	1154 (42.2)	599 (21.9)	2732 (100.0)	
**Sex, *n* (%)**					<0.001
Male	251 (25.6)	342 (29.6)	335 (55.9)	928 (34.0)	
Female	728 (74.4)	812 (70.4)	264 (44.1)	1804 (66.0)	
**Age, *n* (%)**					<0.001
Under 20’s	62 (6.3)	6 (0.5)	9 (1.5)	77 (2.8)	
20’s	183 (18.7)	49 (4.2)	42 (7.0)	274 (10.0)	
30’s	133 (13.6)	98 (8.5)	50 (8.3)	281 (10.3)	
40’s	163 (16.6)	110 (9.5)	69 (11.5)	342 (12.5)	
50’s	140 (14.3)	148 (12.8)	95 (15.9)	383 (14.0)	
60’s	177 (18.1)	336 (29.1)	160 (26.7)	673 (24.6)	
70’s	107 (10.9)	318 (27.6)	134 (22.4)	559 (20.5)	
Over 80’s	14 (1.4)	89 (7.7)	40 (6.7)	143 (5.2)	
**Dietary Supplement Use, *n* (%)**					<0.001
Present	301(30.7)	451 (39.1)	122 (20.4)	874 (32.0)	
Past	298 (30.4)	355 (30.8)	209 (34.9)	862 (31.6)	
Never	125 (12.8)	74 (6.4)	64 (10.7)	263 (9.6)	
Never but Future	255 (26.0)	274 (23.7)	204 (34.1)	733 (26.8)	

*p*-values were calculated χ^2^ test.

### 3.2. Use of Dietary Supplements

Among all subjects, 32.0% were currently using dietary supplements and 31.6% had used dietary supplements in the past (Table 1). Past use of dietary supplements in healthy subjects (30.4%) was similar to present use (30.7%). In contrast, use increased over time in ambulatory patients (from 30.8% to 39.1%) and decreased over time in admitted patients (from 34.9% to 20.4%). However, 20.4% of admitted patients still used dietary supplements. In this survey, we did not ask what kind of dietary supplements they used, but previous reports [9] and some internet surveys showed that various type of dietary supplements including not only vitamins/minerals, but also herbal extracts and its ingredients, were used in Japan.

### 3.3. Awareness of Dietary Supplements

Awareness of dietary supplements is shown in Table 2. In terms of safety, more than 40% of subjects thought that dietary supplements were safe (strongly agree or agree). There were no differences between ambulatory patients and healthy subjects. However, more admitted patients thought that dietary supplements were safe (strongly agree: 24.9%) compared with ambulatory patients (14.1%) and healthy subjects (12.3%). In terms of price, more than 80% of subjects thought that dietary supplements were expensive (strongly agree: 51.1%; agree: 31.3%). However, fewer admitted patients thought that dietary supplements were expensive (strongly agree: 45.9%; agree: 25.6%). In terms of effectiveness, about 40% of subjects thought that dietary supplements were effective (strongly agree: 5.6%; agree: 33.2%). There were no significant differences among three groups. However, admitted patients likely thought that dietary supplements were effective (strongly agree: 9.6%) compared with ambulatory patients (5.4%) and healthy subjects (3.5%).

**Table 2:** Awareness of dietary supplements.

	Strongly Agree	Agree	Neither Agree nor Disagree	Disagree	Strongly Disagree	*p*-value
**Safe (%)**						<0.001
All subjects	15.7	29.5	30.8	17.2	6.8	
Healthy subjects	12.3	30.9	31.3	18.6	6.9	
Ambulatory patients	14.1	28.8	31.6	18.8	6.8	
Admitted patients **^,††^	24.9	28.3	28.5	11.9	6.4	
**Expensive (%)**						<0.001
All subjects	51.1	31.3	12.2	3.4	2.1	
Healthy subjects	49.0	35.4	11.5	2.1	2.0	
Ambulatory patients *	55.5	30.5	8.4	3.8	1.7	
Admitted patients **^,††^	45.9	25.6	20.6	5.0	2.9	
**Effective (%)**						0.232
All subjects	5.6	33.2	28.6	23.2	9.4	
Healthy subjects	3.5	34.9	30.0	22.5	9.1	
Ambulatory patients	5.4	33.3	26.2	25.3	9.8	
Admitted patients	9.6	29.8	30.7	20.6	9.2	
**Substitute for medicines (%)**						<0.001
All subjects	2.0	8.1	14.2	28.3	47.4	
Healthy subjects	1.2	6.4	13.8	27.4	51.3	
Ambulatory patients	1.6	8.7	11.8	29.7	48.2	
Admitted patients **^,††^	4.3	9.8	19.3	27.3	39.2	
**No problem in co-administration with medicines (%)**						<0.001
All subjects	11.9	19.4	22.4	18.8	27.4	
Healthy subjects	7.4	18.1	22.0	22.3	30.1	
Ambulatory patients **	12.4	20.9	21.3	17.7	27.7	
Admitted patients **^,††^	18.6	18.8	25.5	14.8	22.4	

Missing values were excluded; *p*-values were calculated Kruskal-Wallis test; * *p* < 0.0167, ** *p* < 0.0033 *vs.* health subjects, and **^††^** *p* < 0.0033 *vs.* ambulatory subjects by Bonferroni post hoc test.

Overall, most subjects did not believe that dietary supplements were a substitute for medicines, with no differences between ambulatory patients and healthy subjects. In contrast, more admitted patients thought that dietary supplements could be substituted for medicines (strongly agree: 4.3%) compared to ambulatory patients (1.6%) and healthy subjects (1.2%). There was a significant difference among groups in terms of concomitant use of supplements with medicines. Admitted patients (strongly agree: 18.6%; strongly disagree: 22.4%) and ambulatory patients (strongly agree: 12.4%; strongly disagree: 27.7%) were more likely to think that concomitant use of supplements and medicines was safe compared with healthy subjects (strongly agree: 7.4%; strongly disagree: 30.1%).

Current user of dietary supplements thought that dietary supplements were effective (strongly agree: 5.8% healthy subjects, 7.2% ambulatory patients, and 17.7% admitted patients), substitute for medicines (strongly agree: 1.7% healthy subjects, 2.6% ambulatory patients, and 6.1% admitted patients), and no problem in co-administration with medicines (strongly agree: 10.0% healthy subjects, 17.3% ambulatory patients, and 29.3% admitted patients). All of these numbers are higher than those in Table 2. In addition, ambulatory and admitted patients using dietary supplements tended to think that dietary supplements were safe (strongly agree: 12.8% healthy subjects, 16.1% ambulatory patients, and 31.9% admitted patients). However, price (strongly agree: 43.2% healthy subjects, 52.4% ambulatory patients, and 45.3% admitted patients) did not affect dietary supplement use in all groups.

### 3.4. Purpose of Using Dietary Supplements

Purpose of using dietary supplements is shown in Table 3. Among all subjects, maintenance of health and nutritional support were the primary and secondary reasons, respectively, for using dietary supplements; there were no significant differences among groups. More ambulatory patients (33.3%) used dietary supplements to prevent disease compared with admitted patients (23.1%) and healthy subjects (24.9%). Only 3.7% of healthy subjects used dietary supplements to treat their diseases, whereas, 10.0% of ambulatory patients and 13.2% of admitted patients used dietary supplement for this purpose. Use of supplements for beauty purposes was lowest in admitted patients (5.8%) and highest in healthy subjects (23.6%). There was no significant difference among groups; however, 4.4% of ambulatory patients and 4.1% of admitted patients used dietary supplements without any specific purpose.

**Table 3:** Purpose of using dietary supplements.

	Yes	No	*p*-value	Odds Ratio	95% CI
**Maintenance of health (%)**			0.250		
All subjects	70.6	29.4			
Healthy subjects	73.8	26.2		1	
Ambulatory patients	68.2	31.8		0.68	0.48–0.96
Admitted patients	71.9	28.1		0.84	0.51–1.39
**Nutritional support (%)**			0.161		
All subjects	36.7	63.3			
Healthy subjects	40.5	59.5		1	
Ambulatory patients	33.8	66.2		0.86	0.63–1.19
Admitted patients	38.0	62.0		0.95	0.60–1.51
**Prevention of disease (%)**			0.014		
All subjects	29.0	71.0			
Healthy subjects	24.9	75.1		1	
Ambulatory patients	33.3	66.7		1.30	0.92–1.84
Admitted patients	23.1	76.9		0.79	0.47–1.32
**Treatment of disease (%)**			0.001		
All subjects	8.3	91.7			
Healthy subjects	3.7	96.3		1	
Ambulatory patients	10.0	90.0		2.87	1.42–5.78
Admitted patients	13.2	86.8		4.03	1.75–9.28
**For beauty (%)**			<0.001		
All subjects	15.3	84.7		1	
Healthy subjects	23.6	76.4		0.61	0.40–0.93
Ambulatory patients	12.2	87.8		0.29	0.13–0.69
Admitted patients	5.8	94.2			
**Without any specific purpose (%)**			0.911		
All subjects	4.6	95.4			
Healthy subjects	5.0	95.0		1	
Ambulatory patients	4.4	95.6		0.76	0.37–1.56
Admitted patients	4.1	95.9		0.69	0.24–2.04

*n* = 872; Subjects answered dietary supplement use “present”; *p*-values were calculated χ^2^ test; Odds Ratio were calculated logistic regression analysis adjusted for sex and age.

### 3.5. Experience with Beneficial or Adverse Effects from Using Dietary Supplements

Among subjects who used dietary supplements in the past and present, only 23.8% experienced beneficial effects from dietary supplements. However, more admitted patients (28.4%) felt beneficial effects compared to ambulatory patients (25.1%) or healthy subjects (22.1%). On the other hand, 3.3% of subjects experienced adverse effects from using dietary supplements. The most common adverse effects were diarrhea and constipation (28.3%), fatigue (18.3%), allergic reactions (16.7%), abdominal pain (15.0%), vomiting (11.7%), and headache (6.7%).

### 3.6. Concomitant Use of Dietary Supplements and Medicines

Most ambulatory patients (91.3%) and admitted patients (81.8%) took medicines; in contrast, only 10.6% of healthy subject took medicines (Table 4). Furthermore, 36.8% of ambulatory patients, 17.7% of admitted patients, and 4.3% of healthy subjects took dietary supplements and medicines concomitantly.

**Table 4:** Concomitant use of dietary supplements and medicines.

	Medicines *n* (%)	Dietary Supplements *n* (%)	Parallel Use *n* (%)
Healthy Subjects (*n* = 979)	104 (10.6)	301 (30.7)	42 (4.3)
Ambulatory Patients (*n* = 1154)	1054 (91.3)	451 (39.1)	425 (36.8)
Admitted Patients (*n* = 599)	490 (81.8)	122 (20.4)	106 (17.7)

Table 5 shows the number of subjects taking concomitant dietary supplements and medicines. The most common pattern was one kind of dietary supplement and one kind of medicine (*n* = 44). However, six subjects took more than five dietary supplements and more than five medicines concomitantly.

**Table 5:** Number of dietary supplements and medicines used concomitantly.

Number of Dietary Supplements	Number of Medicines				
	1	2	3	4	5≤
1	44	43	27	28	37
2	43	40	33	14	32
3	16	17	13	8	13
4	4	4	3	2	4
5≤	10	5	3	3	6

*n* = 452; Missing values were excluded.

### 3.7. Declaration of Dietary Supplements Use to Primary Care Doctors

In patients who took dietary supplements and medicines concomitantly, only 30.2% of admitted patients and 28.0% of ambulatory patients declared their use of dietary supplements to their attending physicians. In other words, almost 70% of patients used dietary supplements on their own, without consulting physicians. Table 6 shows reasons for no declaring dietary supplements use to physicians in each ambulatory and admitted patient.

**Table 6:** Reasons for not discussing dietary supplement use with physicians.

Reasons	Ambulatory (*n*)	Admitted (*n*)
Dietary supplements that they use does not relate to their treatment	25	2
Doctors might deny dietary supplements use	19	2
Doctors never ask about dietary supplements use	14	5
Dietary supplements are just food	16	2
No need to say	7	6
There are any influences to medication (self-judgment)	8	1
There are not any opportunities to tell	5	1
Doctors do not have any knowledge about dietary supplements	3	1
There are not any problems in using dietary supplements	3	0
Use dietary supplements only as needed	3	0
Other	12	4

*n* = 112 in ambulatory patients and *n* = 24 in admitted patients; Subjects answered this question.

## 4. Discussion

In this study, we clarified that not only ambulatory patients but also admitted patients used dietary supplements, and they used it for treatment their diseases. These patients also took medicines concurrently without consulting physicians.

In the United States, 48.8% of people used dietary supplements from 2007 to 2010 [10]. Previous reports show that use of dietary supplements in Japan has increased over time from 10.9% in 2001 [11], 11.0% in males and 16.4% in females in 2003 [12], and 45.8% in older adults in 2008 [9], even if factors such as sex, age, socioeconomic status, and health-related characteristics are known to affect use of dietary supplements [10,13,14,15,16]. In addition, recognition also affects dietary supplement use. Dietary supplements were not regulated in Japan. Most dietary supplements are the form of capsules, tablets, powders, or liquid, and some are the form of regular foods in Japan. In this situation, some people take dietary supplements without consideration for the risk of them. In these days, as dietary supplement use increases, associated health problems also increase. Health problems associated with dietary supplement use have two causes. One is use of low quality or illegal products that contain drug ingredients [17,18]. To avoid health problems caused by these products, the Japanese government constantly surveys and checks these products on websites and retail stores. Another is inappropriate use of dietary supplements, including excessive intake and concomitant use of various dietary supplements and/or medicines. In particular, inappropriate use of dietary supplements in patients may be associated with severe health problems. To avoid health problems caused by inappropriate use, communication between patients and physicians are important.

It is recognized that infants, children, pregnant women, the elderly, and patients are susceptible to dietary supplements. It is important to identify dietary supplement use in these high-risk groups and to stop inappropriate use. Inappropriate use of dietary supplements by Japanese children [19] and pregnant women [20] has been defined. In Japan, most children have a good nutritional state and thus do not require dietary supplements. On the other hand, folic acid supplements are recommended for pregnant women because it is difficult to obtain adequate amounts of folic acid from food [21]. However, we confirmed that pregnant women could not avail of dietary supplements appropriately [20]. Aside from children and pregnant women, many older people in Japan appear to use dietary supplements; some of them use dietary supplements for treatment of diseases [22]. In this study, we investigated the awareness and use of dietary supplements among Japanese subjects.

Our results showed that 36.8% of ambulatory patients and 17.7% of admitted patients took dietary supplements with their medicines and thought that this practice was safe. However, many reports indicate that dietary supplements interact with medicines. The most well-known example is St. John’s wort (*Hypericum perforatum* L.). St. John’s wort contains hyperforin, which increases the expression of cytochrome P450 (CYP), especially CYP3A4, and affects drug metabolism in the liver [23]. Other herbs (e.g., black cohosh, coleus forskohlii, echinacea, garlic, ginkgo, ginseng, green tea, kava, and milk thistle) [24,25,26,27,28] and ingredients (e.g., catechins [29], curcuminoids [30], isoflavones [31], quercetin [32], polyphenols [33], and resveratrol [34]) also affect drug metabolizing enzymes.

To avoid interactions between prescription medications and dietary supplements, physicians need to know whether their patients use dietary supplements or not. However, as shown in this survey, most patients do not discuss these supplements with their physicians, which is consistent with previous reports [35]. One reason for this lack of discussion is that most physicians do not ask about dietary supplement use, probably because the consultation time for each patient is limited. In addition, 5 admitted patients answered “Doctors never ask about dietary supplements use” (Table 6), it means that some of physicians did not care whether their patients used dietary supplements or not. It might be caused by insufficient recognition of dietary supplements. At the same time, most patients do not think that dietary supplements will affect their medication. Thus, both patients and physicians do not fully recognize the risk of interactions between dietary supplements and medications [36]. It is also reported that both of patients and physicians are poorly understood the regulation of dietary supplement in the USA [37], even though dietary supplements are regulated by the U.S. Food and Drug Administration (FDA) under Dietary Supplement Health and Education Act. As dietary supplements are not as safe as they believe [38], education for both physicians and patients is important in order to avoid health problems associated with dietary supplements.

Consistent with a previous internet survey in Japan, 3.3% of all subjects experienced adverse effects by using dietary supplements, even if most cases were not severe. In this survey, we did not ask which type of product was used. Thus we could not determine any relationship between dietary supplements and adverse effects. However, many subjects used several dietary supplements and medicines concurrently. Even if we asked which type of product was used, it would be impossible to determine the cause of health problems. To avoid unexpected health problems caused by dietary supplements, patients should not use dietary supplements for disease treatment or concurrently with medicines without consulting by physicians.

There are some limitations in this study. The number of admitted patients was lower than the number of ambulatory patients or healthy subjects, because cooperation with primary doctors was essential to conduct this survey in admitted patients. In addition, we did not ask type, periods, and frequency of dietary supplements use or medications. So, we could not evaluate the exact risk of concomitant use of dietary supplements and medicines in this study. Further investigations are needed.

## 5. Conclusions

We clarified that most patients used dietary supplements without consulting physicians, and some of them experienced adverse effects from using dietary supplements. To avoid health problems, it is important that physicians ask patients about dietary supplement use and those patients should inform their physicians about these supplements if physicians do not ask.
